# The National Insurance Act and the Voluntary Hospitals

**Published:** 1912-02-03

**Authors:** 


### THE NATIONAL INSURANCE ACT AND THE VOLUNTARY HOSPITALS.

### Conference, Opinions, Policy and Security.

Sufficient time has now elapsed to have enabled hospital managers to peruse and consider the Insurance Act and its probable effect upon the voluntary hospitals. The newspapers have contained numerous statements from various hospital sources as to the effects this Act has had upon contributions to hospitals by the public. The net result is that probably every voluntary hospital has received clear intimation that the effect of the Act will be to diminish and not to increase the volume of public support for voluntary hospitals. It is early days, however, to attempt to realise in pounds sterling the actual diminution in revenue which is likely to result. The only reliable estimates are those which give the percentage of the present ordinary income of each group of hospitals throughout the United Kingdom which can be affected adversely. Apart from legacies, these percentages are: in London 23 per cent., in the provinces and Wales 47½ per cent., in Scotland 45 per cent., in Ireland 29 per cent. It is only, however, when legacies are added that the grievous crippling of the finances of our voluntary hospitals can be fully realised. Taking Scotland, for instance, it is not too much to say that probably 75 per cent. of the whole revenue of the voluntary hospitals in that country is liable to be diminished or destroyed. It is typical of the paralysed condition of the House of Commons under its present managers that no real discussion took place in regard to hospital benefit and the effect of the Act upon our voluntary hospitals. Indeed when the clauses of the Act which affect hospitals were before the House they were rushed through without discussion, although a considerable number of members had put down amendments, and had waited hours in the hope of being able to discuss the question of hospital benefit fully and on its merits. These facts should be borne in mind, because they must have a material bearing upon the claim which the voluntary hospitals undoubtedly have to secure an amendment of the existing Act without a moment's avoidable delay.

### The Question of Out-Patients.

A meeting has been summoned on February 1 by Mr. Sydney Holland of representatives of the London hospitals, presumably to discuss the questions raised in his letter to the newspapers some ten days ago. We have reason to think it will be found that, whereas there are various and diverse opinions as to in-patient benefit, the overwhelming majority of hospital managers who know their work are of opinion that the present out-patient department as it exists to-day must be abolished. We have gratifying evidence of the crystallisation of hospital opinion in the following resolution, which has been unanimously passed at a recent meeting of the Council of the British Hospitals Association:—

"The British Hospitals Association is strongly of opinion that upon the Insurance Act coming into force, insured persons, except for accidents or sudden emergency, should no longer be received in the out-patient or casualty departments, unless accompanied by a certificate or introduced personally by the medical practitioner who is in attendance, and in such a case after consultation he (the patient) should be referred back to the same medical attendant, with an expression of the opinion of the hospital physician or surgeon on the case.

"And that a list of all such insured persons and the practitioners by whom they are sent should be forwarded to the Insurance Committees of the district periodically."

### Hospital Abuse and Malingering Prevented.

We welcome this resolution because it indicates clearly that hospital managers, wholly distinct from members of the medical profession, are prepared to co-operate with the Government under the National Insurance Act to prevent hospital abuse and malingering on the part of everybody concerned by rendering it impossible for practitioners on the panel to throw any burden of sick attendance upon the hospitals which they themselves ought properly to bear, and at the same time to co-operate with the medical profession by making arrangements for a consultation branch at the voluntary hospital, to which every medical practitioner will have free access when occasion may arise. The resolution further proves that the hospital authorities are ready to co-operate with the Insurance Committee of each district with the view of preventing any malingering, so that the Treasury, as well as the patients and the hospitals, may all be adequately protected by working in harmonious co-operation. We hope that this action on the part of the representatives of the voluntary hospitals may induce those responsible to provide promptly that the maintenance of these institutions in ever-increasing efficiency shall be aided by the Government agreeing to amend the National Insurance Act on the lines and to the extent suggested in The Hospital of December 9 and 16, pages 253 et seq. and 288.

### Insured Persons and In-Patient Benefit.

Our readers must be prepared for considerable difference of opinion amongst metropolitan hospital managers in regard to the treatment of insured persons as in-patients. We have tried to make it clear on more than one occasion that, if the managers are prudent and will exhibit a little statesmanship, they ought to be able to secure not only the continuance of our excellent system of voluntary hospitals, but its improvement and extension, so as to make it adequate to meet the requirements of all classes, members of which may at any time need hospital care and treatment. This can be readily done, providing they appreciate that so long as they do not receive State aid, but are in fact self-contained voluntary institutions, the question of their supersession by State hospitals is never likely to arise. The voluntary hospital will always have its proper work to do. The Insurance Act leaves to the hospitals all the dependants—that is the wives and families of insured persons—as well as all the poorer members of the community, who alone constitute an army more than large enough to require for their adequate care and treatment all the hospital beds which the voluntary hospitals at present maintain. It is quite true that the Insurance Act as it stands may prove a curse and not a blessing to the working classes of the United Kingdom unless some means are promptly found whereby hospital benefit, when it is required, can be assured to the workman, being an insured person. The Act as it stands, if it is not speedily amended, is calculated to cause grievous riots like that which has taken place this week in Germany. The cause may be the discovery by each workman that deductions are made each week from his wages to pay for his adequate medical attendance, while he cannot properly demand the hospital care he needs as a free gift in future from the voluntary hospitals. As an insured person he at once comes under the care of the State, and it is the State's duty to provide him with hospital benefit as a part of that adequate medical treatment of which our politicians have talked so glibly and with so little understanding during recent months.

### Questions of Policy.

The position then in a nutshell is this: The voluntary hospitals are prepared to continue to do their work of granting free medical relief to all who may be entitled to receive such benefits at their hands. The working man's ineligibility as an insured person to seek and obtain such medical relief at the voluntary hospitals does not arise from any fault or act of the voluntary hospitals, but from the act of the Government, and it is to the Government the insured must look for protection from the dangers which must result to the breadwinners of this country if they are to continue to be deprived of the hospital benefit and care which a large percentage of them must need each year in the ordinary course. We have therefore the State on the one hand and the voluntary hospitals on the other, with the responsibility which devolves upon each pretty clearly defined. The State has taken upon itself the duty of providing hospital benefit for the breadwinners of this country from July 15, 1912 (or some subsequent date not being later than January 1, 1913), and it is for the State to approach the voluntary hospitals and to enter into an agreement with the managers of each to admit insured persons as in-patients on the State's undertaking to defray the *pro rata* cost of their treatment and maintenance during their stay in the hospital, plus 10 per cent. for medical attendance. The acceptance of these cases under these conditions will mean, no doubt, a new class of work, but it will be work which will extend the sphere of influence and usefulness of the voluntary hospitals, and may therefore be properly undertaken by the managers. Such an agreement between the State and the hospital managers will in no sense interfere in any way with or affect the voluntary character of the voluntary hospitals. Their voluntary work will go on in those portions of the hospital which are set apart for the reception of free patients. The State cases will be received and treated in wards or pavilions specially set apart for the reception of State cases. This system has been carried out with readiness and success on the Continent of Europe and in other countries, the free cases and many of the State cases, coming as they do from the same class, being often treated in the same wards.

### The Voluntary Principle Maintained.

Those who refer to The Hospital of December 9 and 16, pages 253 and 288 respectively, will see that the proposed *pro rata* plan with loans will preserve the voluntary hospitals and strengthen them. A similar plan has been adopted in London by the Hospital Saturday Fund, which sends patients to voluntary hospitals under it, to the satisfaction of all concerned. There is no difficulty in practice in fixing the *pro rata* cost of a patient, and classification will entirely prevent a voluntary hospital from becoming an institution for insured persons only. On the contrary, each hospital that adopts this plan would increase its popularity and usefulness and safeguard its interests to the fullest extent. Under the *pro rata* system with loans full provision will be made to cover additions, improvements, new buildings, and the setting-up of new departments as and when required. *Pro rata* payments will not involve State representation in the management, but merely State inspection of the wards or pavilions in which State patients are received. The question of payment for the medical treatment of the insured when in a voluntary hospital is also satisfactorily provided for, so that the objections which some have thought to attach to the *pro rata* system do not in fact exist. On the other hand, the suggestions that the State might make grants in aid of large sums to meet deficiencies occurring in particular years is not business. It is not a suggestion which the State is likely to further, and it has the great objection that if the State agreed to make large grants in aid it would certainly be entitled to enforce representation in the management, because such grants would entitle it to such representation in the same way as all other contributors. It would lead to constant friction between the State and the management, because the temptation would be to incur expenditure in various directions on the principle that the responsibility of providing the money would devolve upon the State, so that the total of any lump sum would soon come to be regarded as of relatively small importance. There is therefore a very grave danger that grants in aid would lead to extravagance in the management, to friction between the Government and the managers, to State representation in the management, and ultimately to the extinction of the voluntary hospitals and the substitution for them of State hospitals all over the country. The *pro rata* plan safeguards the hospitals against all these dangers. It is sound business, and it is a method which is calculated to commend itself to the Government, because it would entail a minimum expenditure out of State funds and a maximum hospital provision for all classes of the community.

